# *Caulobacter* and *Novosphingobium* in tumor tissues are associated with colorectal cancer outcomes

**DOI:** 10.3389/fonc.2022.1078296

**Published:** 2023-01-27

**Authors:** Bin Zhou, Linli Shi, Min Jin, Mingxia Cheng, Dandan Yu, Lei Zhao, Jieying Zhang, Yu Chang, Tao Zhang, Hongli Liu

**Affiliations:** Cancer Center, Union Hospital, Tongji Medical College, Huazhong University of Science and Technology, Wuhan, China

**Keywords:** microbiomes, colorectal cancer, outcomes, 16sRNA, *Caulobacter*, *Novosphingobium*

## Abstract

Diversity and composition of the gut microbiome are associated with cancer patient outcomes including colorectal cancer (CRC). A growing number of evidence indicates that *Fusobacterium nucleatum* (Fn) in CRC tissue is associated with worse survival. However, few studies have further analyzed the differences in bacteria in tumor tissues of different patients depending on the survival time of CRC patients. Therefore, there is a need to further explore the bacterial differences in tumor tissues of patients with different prognoses and to identify key bacteria for analysis. Here, we sought to compare the differences in tumor microbiome between patients with long-term survival (LS) longer than 3 years or 4 and 5 years and patients with short-term survival (SS) in the present study cohort. We found that there were significant differences in tumor microbiome between the LS and SS and two bacteria—*Caulobacter* and *Novosphingobium*—that are present in all of the three groups. Furthermore, by analyzing bacteria in different clinical features, we also found that lower levels of microbiome (*Caulobacter* and *Novosphingobium*) have long-term survival and modulating microbiome in tumor tissue may provide an alternative way to predict the prognosis of CRC patients.

## Introduction

Colorectal cancer (CRC) continues to be the third leading malignant cancer and the fourth leading cause of cancer-associated mortality globally. It is estimated that more than 1,800,000 new cases of CRC will be diagnosed worldwide per year, resulting in over 880,000 deaths (1). With traditionally multimodal treatments that included surgery, radiotherapy, chemotherapy, and immunotherapy (2), some patients have long-term survival, while others do not, and the reason was unclear.

More than 30 trillion microbiomes inhabit the human intestinal tract and play an important role in health and disease conditions, including cancer. Among the entire gut microbiome, there is a group of natural bacteria with anti-tumor properties called probiotics (3). There also exists a part of bacteria that can promote the development of tumors in the body and lead to poor prognosis of tumors, such as Fn and *Helicobacter pylori* (4, 5). A growing body of evidence suggested a potential link between the microbiota and colorectal carcinogenesis, immune modulation of the tumor microenvironment, and response to immunotherapy (6). Overwhelming evidence indicates relationships between bacteria and cancer outcomes. For example, *Fusobacterium nucleatum* in CRC tissue display distinct features, including high-level microsatellite instability (MSI), low-level CD3+ T-cell density, and worse survival, and it can influence outcomes through diverse ways (7, 8). Our incomplete knowledge of the interactions between microbes, distinctive tumor features, and the host immune system highlights the critical need for transdisciplinary integrated analyses of microbiomes and cancer. The gut microbiome can secrete toxins, chronic inflammation mediators, and metabolites, or interact with epithelial cells to promote tumor progress. Similarly, it also affects the therapeutic effect of tumors through various ways, such as surgery, immunotherapy, chemotherapy, and even radiotherapy (9, 10).

The latest research has found that bacteria have a direct relationship with tumor prognosis. For example, in pancreatic cancer, it is found that the tumor microbiota of patients with a survival period of more than 5 years is significantly different from that of patients with a survival period of less than 5 years, indicating that microbiomes may be used as markers to predict the prognosis of patients with pancreatic cancer (11). In cervical cancer, it was found that the diversity of gut microbiota was related to the efficacy of radiotherapy and chemotherapy. In addition, differences in the composition of different microorganisms are associated with short-term and long-term survival. The short-term survival fecal samples were significantly enriched in porphyrinomonas, porphyrinomonaceae, and dialysis bacteria, while the long-term survival fecal samples were significantly enriched in Shigella, Enterobacteriaceae, and Enterobacteriaceae (12). Also in CRC, *F. nucleatum* is an anaerobic Gram-negative pathogen whose enrichment in CRC tissues is associated with shorter survival and serves as a poor prognostic biomarker (13). Although numerous studies have explored CRC and microbes and find the effect of Fn bacteria on CRC, not all short-term survival cancer patients have Fn enrichment, and the composition of the human CRC microbiome that contributes favorably or adversely to outcomes of CRC remains incompletely studied. Therefore, it is necessary to further study the relationship between tumor microbiomes and the prognosis of CRC patients to find out the pathogenic or dominant microbiota.

In this study, we hypothesized that tumor tissue bacteria could affect the prognosis of CRC. The microbial community and clinical characteristics of patients with different outcomes were compared, and key bacteria were found for analysis. Analyzed with different clinical characteristics to clarify that the microbiota in tumor tissue can have an impact on the survival of patients, it provides new ideas for finding new predictors and therapeutic targets in the future.

## Materials and methods

### Patients and sample collection

The participants enrolled in this study were Chinese patients from 1 January 2015 to 1 November 2017 at the Union Hospital of Tongji Medical College, Huazhong University of Science and Technology, Central China. Clinical variables, demographics, and pathologic reports of the participants were collected from hospital electronic medical records. Tumor tissues were obtained from patients diagnosed with primary CRC who have undergone surgical treatment. Patients treated with chemotherapy, radiotherapy, or antibiotics before surgery were also excluded from this study. A total of 318 tumor tissues were collected. Samples of tumor mucosa tissue were fixed in formalin and embedded in paraffin (FFPE). Three sections of 5-µm FFPE of CRC tissue were placed in sterile microtubes and then stored at room temperature until use for 16S rRNA MiSeq sequencing. All subjects provided written informed consent before they participated in the study. The Ethics Committee of Tongji Medical College of Huazhong University of Science and Technology approved this study (No. 2014-041 and No. 2018-S377).

### DNA extraction and 16 S rRNA gene sequencing

Frozen tissues were cut into small pieces and homogenized using a handheld homogenizer for 30 s in 100 μl of C1 buffer using a PowerSoil DNA Isolation Kit. DNA extraction using the Omega Mag-Bind Soil DNA Kit from whole-tissue sections of CRC FFPE tissue blocks and purification were performed using the QIAamp DNA Mini Kit. The V3–V4 region of the 16S rRNA gene was amplified by PCR and the following primers: initial denaturation for 2 min at 98°C; 30 cycles of 15 s at 98°C, 30 s at 55°C, and 30 s at 72°C; and final extension for 5 min at 72°C. All purified amplicons for each sample were mixed. DNA library was constructed according to the TruSeq Nano DNA LT Library Prep Kit, and sequencing was performed on the Illumina MiSeqPE250.

### Sequence and statistical analyses

Patient demographic and clinical information was compared using chi-squared test. Data analysis was performed with GraphPad Prism 8 software (GraphPad Inc., San Diego, CA, USA) and IBM SPSS Statistics 22.0 software (IBM Inc., Armonk, NY, USA). The richness and evenness of the species were performed by R software and were represented on a rank abundance curve. Microbial alpha diversity was analyzed by a sampling-based OTU table. Microbial alpha diversity was presented by Chao1, ACE, Shannon, and Simpson diversity indices (Paul et al., 2015), which were calculated using the Wilcoxon rank sum test. *t*-test was used to compare alpha diversity and tumor characteristics in patients with proximal and distal CRCs. Using linear discriminant analysis effect size (LEfSe) can show differentially abundant taxa between groups. To determine the primary bacterial differences between the two groups and discriminate biomarkers, the threshold of the logarithmic linear discriminant analysis (LDA) score was set to 2. The LDA effect size method was used to distinguish the characteristics of different microorganisms in the microbiota. Lefse uses the Kruskal–Wallis test to detect features with significantly different abundances among specified taxa, while LDA is used to evaluate the impact of each feature. The expression level of *Caulobacter* and *Novosphingobium* was categorized into “high” and “low” using the median value as the cutoff point. Fisher’s exact test was used to evaluate the relationship between bacteria and tumor characteristics. Overall survival (OS) was defined as the time from diagnosis to death from any cause. Patients who did not experience death were censored at the date of last follow-up. The Kaplan–Meier method was used to analyze the relationship between differential bacteria and OS in CRC patients. The Log-rank test was used to test the difference in survival distributions between subgroups. Receiver operator characteristic (ROC) and area under the curve (AUC) analysis were performed using Spss22.

## Results

### Tumor microbiome communities are different between LS and SS

**Figure 1:**
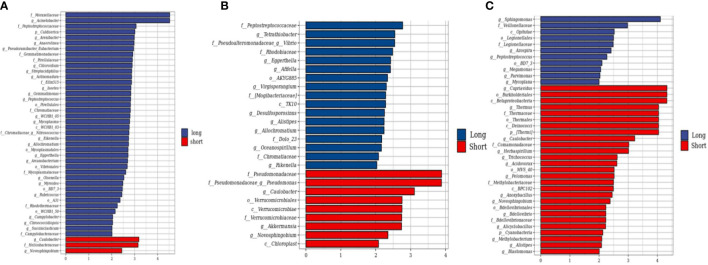
Compositional differences of the gut microbiome in short and long-term survivors. **(A)** The different abundance of bacterial taxa between the patients who survived more than 5 years and shorter were identified by LEfSe. The histogram showed that the LDA scores of taxa were differentially abundant between the two groups. Three bacteria enriched in the short-term survivor. **(B)** The LEfse analysis of patients who survived more than 4 years. **(C)** The LEfse analysis of patients who survived more than 3 years.

A total of 318 CRC tumor tissues from Chinese patients with a mean age of 56.9 years were collected. Bacterial DNA was extracted from 318 surgically resected CRC tumor, and taxonomic profiling *via* 16S RNA Illumina MiSeq sequencing was performed. To explore the role of the human tumor microbiome composition in mediating clinical outcomes of CRC patients, we compared the patients who survived more than 5 years and the short-term survivors who survived less than 5 years. We found the differences in the microbial composition of tumor tissues between the LS and SS groups, with three bacteria—*Caulobacter*, Helicobacteraceae, and *Novosphingobium*—enriched in the SS group compared to the LS group (**Figure 1A**). Then, we use the same method to detect the microbiome between the patients who survived more than 3 years or 4 years and shorter. Similarly, in comparing patients surviving more than 3 or 4 years, *Caulobacter* and *Novosphingobium* were enriched in the tumor tissues of patients with short OS (**Figures 1B, C**). We therefore hypothesized that the enrichment of both bacteria in tumor tissues may affect patient survival time.

### Tumor microbial communities are associated with outcomes in CRC patients

**Figure 2:**
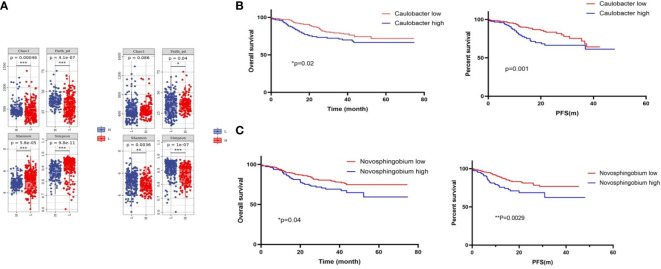
Tumor microbial diversity influences the outcome of CRC patients. **(A)** Alpha diversity box plot (Chao1, Shannon, Simpson, and Faith-pd) of CRC patients. **(B)** Kaplan–Meier curves for colorectal cancer overall survival according to the relative status of *Caulobacter* in CRC tumor tissues and the relative status of *Novosphingobium* **(C)**. Cases with bacteria were categorized as high or low/negative using the medium value as the cutoff point. The *p*-value was calculated by the log-rank test. CRC, colorectal cancer.

To explore the role of these two bacteria in mediating clinical outcomes of CRC patients, we next stratified all patients into a bacteria-high group (BH) and a bacteria-low (BL) group based on their median relative abundance of these two taxa (*Caulobacter* and *Novosphingobium*). Firstly, we analyze the clinical characteristics of the two cohorts. It was found that patients in BH and BL groups were matched with respect to age, gender, stage, and tumor differentiation (**Tables 1**, **2**). In order to investigate intra-tumor bacterial diversity and richness within the samples, we analyzed the alpha diversity indices, including Chao1, Shannon, Simpson, and Faith-pd. It was found that there were significant differences between the low and high bacterial group (*p* < 0.05) (**Figure 2A**). We found that, compared with BL, BH had lower alpha diversity.

**Table 1:** Clinical characteristics of patients with high and low *Caulobacter*.

Characteristics	Caulobacter high, n= 120 (%)	Caulobacter low, n = 198 (%)	p-Value
Age (years)	57.98 ± 14	55.67 ± 12.46	0.17
Gender			0.21
Female	56 (47)	90 (45)	
Male	64 (53)	108 (55)	
pT stage			0.50
T1–2	16 (13.3)	15 (7.6)	
T3–4	92 (76.7)	172 (86.8)	
Tx	12 (10)	11 (5.6)	
pN stage			0.48
N0	60 (50)	108 (54)	
N1–2	60 (50)	90 (46)	
Distant metastasis			0.17
M0	90 (75)	161 (82)	
M1	30 (25)	38 (18)	
Vessel carcinoma embolus			0.72
Yes	30 (25)	46 (23.2)	
No	90 (75)	152 (76.8)	
Neural invasion			0.53
Yes	33 (27.5)	61 (30.8)	
No	87 (72.5)	137 (69.2)	

pT, depth of tumor invasion; M, distant metastasis of primary tumor.

**Table 2:** Clinical characteristics of patients with high and low *Novosphingobium*.

Characteristics	Novosphingobium high, n = 109 (%)	Novosphingobium low, n = 209 (%)	p-Value
Age (years)	57.98 ± 14	55.67 ± 12.46	0.17
Gender			0.17
Female	56 (51.4)	83 (39.7)	
Male	63 (48.6)	126 (60.3)	
pT stage			0.70
T1–2	7 (6.4)	14 (6.7)	
T3–4	97 (89)	187 (89.5)	
Tx	5 (4.6)	8 (3.8)	
pN stage			0.058
N0	49 (45)	118 (56.7)	
N1–2	60 (55)	91 (43.3)	
Distant metastasis			0.85
M0	86 (79)	166 (79.8)	
M1	23 (21)	43 (20.2)	
Vessel carcinoma embolus			0.61
Yes	26 (23.8)	55 (26.4)	
No	83 (76.2)	154 (73.6)	
Neural invasion			0.45
Yes	37 (29.3)	62 (29.8)	
No	72 (70.7)	147 (70.2)	

Next, we analyzed the survival times between the BH and BL in *Caulobacter* through Kaplan–Meier analysis. It was revealed that the OS time and progression-free survival (PFS) time of the BH was shorter than that of BL (*p* < 0.05) (**Figure 2B**). This was consistent with a previous conclusion that *Caulobacter* was enriched in patients with a shorter survival time. Based on these results, we then analyzed *Novosphingobium* using the same method, and we received the same results (**Figure 2C**). Our findings indicated that these two bacteria could affect the survival outcomes in CRC patients, and the low taxa of the bacteria had a better prognosis, suggesting the potential relevance of the microbiome composition in mediating CRC progression. Therefore, we preliminarily concluded that the presence of the two bacteria may have some correlation with the OS and PFS of CRC; the OS and PFS are better in the high group than in the low group.

**Figure 3:**
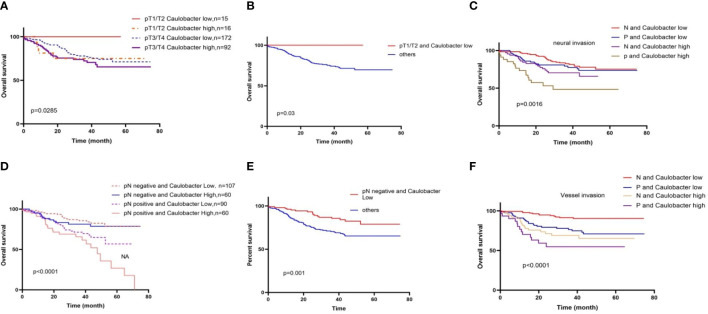
Different clinical characteristics in BL and BS result in distinct overall survival. **(A)** Overall survival stratified by gut diversity and T stage; in the T1/T2 stage, BL groups prolonged the OS. **(B)** BL-T1/2 was significantly better than that of other groups. **(C)** Overall survival stratified by gut diversity and N stage. **(D)** BL-N(−) was significantly better than that of other groups. **(E)** BL outcome was better than BH in negative (N) or positive (P) neural invasion; the double-negative group had a long-term survival compared to other groups. **(F)** BL outcome was better than BH in negative (N) or positive (P) vessel invasion.

Considering the relationship between intra-tumor microbial communities and the outcomes of CRC, we next sought to focus on combined bacteria with clinical features to analyze the effect of bacteria on the survival of CRC patients in different clinical subgroups. Firstly, we assessed the combination of bacteria and tumor T stage, classified bacteria into BH-T1/2, BH-T3/4, BL-T1/2, and BLT3/4. Then, we analyzed the effect of two bacteria on patient time in different tumor sizes. In the *Caulobacter* group, BL-T1/2 was significantly better than that of other groups (**Figures 3A, B**). Moreover, we continued to analyze the N stage of tumor with bacteria, and it also found that the BL-N1/2 group had the best prognosis, while the BH-N3/4 group had the worst outcome (**Figures 3C, D**). Moreover, we also analyzed the prognosis of patients with vascular cancer thrombus or nerve invasion combined with CRC, as previously performed; the double-negative group had long-term survival compared to other groups, especially the double-positive group (**Figures 3E, F**). However, in the *Novosphingobium* group, we never found a significant difference in prognosis between the BL and BS with distinct clinical characteristics (**Figure S1**).

**Figure 4:**
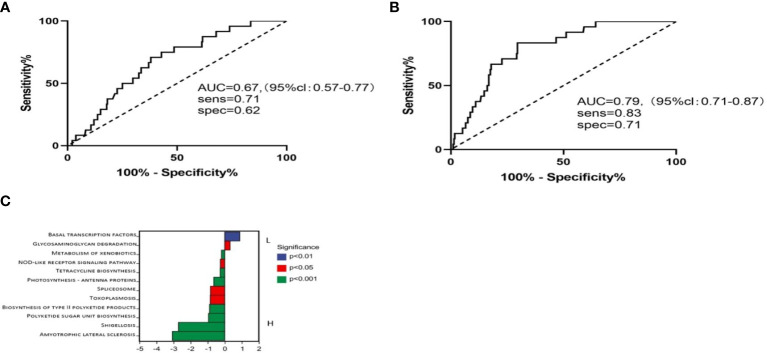
Using these two genera with low abundance to run receiver operator characteristic (ROC) analysis. **(A)** ROC analysis of bacteria abundance as predictive of CRC outcomes. **(B)** ROC analysis of bacteria with different clinical stages. **(C)** Metabolic pathways in BL and BH.

We then used these two genera to predict CRC patients who would survive over 5 years. We found that the combination of these two bacteria resulted in an AUC of 67.4, and if we combined the clinical stage, the AUC increases to 79.2 (**Figures 4A, B**). These data suggested that the presence and abundance of these two taxa communities, *Caulobacter* and *Novosphingobium*, could influence and predict long-term survival in CRC patients.

### Microbiome communities from BL and BH are associated with different metabolic pathways

It has been demonstrated that microbiota imbalances can induce systemic metabolic alterations (14, 15). Conversely, metabolic dysfunction can also induce microbiome imbalances (16). Based on this, we next assessed the metabolic pathways between high and low *Caulobacter* patients. PICRUSt2 software was used to predict the functional composition of the sample by the abundance of the marker gene sequence. The KEGG enrichment analysis results showed that the BL cases exhibited enrichment in the pathways related to basal transcription factors and glycosaminoglycan degradation. In contrast, the BH cases demonstrated enrichment in amyotrophic lateral sclerosis, shigellosis, polyketide sugar unit biosynthesis, toxoplasmosis, spliceosome, photosynthesis, and biosynthesis (**Figure 4C**). The composition of the tumoral microbiome determines a differential enrichment of metabolic functional pathways between LS and SS cases, which may influence patient survival.

## Discussion

This study tested the hypothesis that tumoral microbial communities for CRCs were heterogeneous and tumor bacteria is associated with survival outcomes. Through 16S rRNA high-throughput sequencing of 318 pairs of tumor tissues, we found that CRC patients with a longer survival had significantly different tumor bacteria compared to that of patients with a shorter survival. Furthermore, a distinctive tumor microbiome signature with specific bacterial genus between LS and SS was predictive of survival time. Notably, we demonstrated two specific microbiotas that were associated with CRC outcomes. Studies have shown that *Novosphingobium* was increased in cholangiocarcinoma and lung cancer (17, 18). In our study, we found that *Novosphingobium* was associated with CRC outcomes. Previous studies suggested that the prognosis of CRCs is closely related to the gut microbiome, and patients with different prognoses have different bacteria. Certain specific bacterial compositions could cause better or worse prognosis in patients (19). Our study indicates that patients with short- and long-term survival were colonized with different microbiota, and some bacteria result in worse outcomes. *F. nucleatum* is one of the most well-known bacteria and has the closest relationship with CRC patients. Recent studies have provided varied mechanisms between *F. nucleatum* and colorectal tumor progression, immune microenvironment, treatment, metastasis, and prognosis (20, 21). Mima et al. showed that compared to *F. nucleatum-*negative cases, *F. nucleatum-*low cases and *F. nucleatum-*high cases had a higher mortality rate and the amount of *F. nucleatum* was associated with MSI-high (22).

Recently, there are increasing studies on probiotics, and a large number of data show that probiotics can promote anti-tumor immunity of CRC patients and improve the efficacy of tumor immunotherapy (23). The main mechanisms include the activation of the immune system, the inhibition of carcinogens and carcinogenic agents, and the effect on apoptosis and value-added cells (24). Moreover, studies have shown that probiotics can reduce the complications of chemotherapy, radiotherapy and surgery for CRC, and decrease the mortality rate of patients (25). Microbiome also affects the prognosis of other tumors. Riquelme et al. showed that the tumor microbiome of patients with long-term pancreatic cancer was significantly different from that of patients with short-term pancreatic cancer, and the patients with long-term survival had higher alpha diversity. The feasibility of bacteria in predicting the prognosis of pancreatic cancer was determined, and the possible mechanism was the recruitment and activation of CD8 T cells into the tumor environment (11). In addition, the gut microbiome can have an influence on the outcome of advanced non-small cell lung cancer patients who received immune checkpoint inhibition. A prospective study found lower alpha diversity in patients with lower OS. Ruminococcaceae and Clostridiales were found to be significantly enriched in patients with OS >12 months (26). Similarly, Takada et al. showed that probiotics improved the efficacy of immunotherapy for non-small cell lung cancer, and that patients who used probiotics had a longer PFS (27). In the present study, a higher amount of tissue *Caulobacter* and *Novosphingobium* was associated with worse clinical outcome, and a higher T stage, N stage, or vessel/nerve invasion results in an even worse outcome. These findings suggest a strong positive correlation between the gut microbiome and clinical outcomes in patients with cancer.

There are many factors that affected the prognosis of tumors. Recently, studies show that the gut microbiome can affect the efficacy of surgery, chemotherapy, radiotherapy, and immunotherapy of tumors through various ways, and can also affect the immune microenvironment of tumors. All of the above factors may affect the survival time of patients. Among various mechanisms, the metabolites of the bacteria are one of the important factors. Studies have shown that the metabolites of microorganisms can affect the progression and treatment of tumors and have an impact on the prognosis of tumors (28). Ternes et al. revealed that Fn is able to promote the invasion of CRC cells by producing large amounts of formate, thus leading to the metastatic spread of tumors. The mechanism may be related to the activation of the AhR pathway (29). Glycosaminoglycans are heteropolysaccharides, long-chain polymers without branches, and are composed of an amino sugar (d-glucosamine that is N-acetylated, or N-sulfated, or N-acetyl-d-galactosamine) and either uranic acid (d-glucuronic acid or l-iduronic acid) or galactose. GAGs play a major role in physiological activity in the body, and the GAGs that interacted with growth factors, cytokines, and growth factor receptors are associated with cancer growth and progression (30). The GAGs are involved in signaling cascades, regulating angiogenesis, invasion, and metastasis of malignant cells. Our study indicates that in BL patients, the GAG degradation pathway is apparently higher than BH. This could be a potential mechanism for longer survival time.

The strength of this article is that we collected tissues from 318 CRC tumor patients. We identified differences in the microbial composition of the tumor microenvironment in patients with different survival times. Moreover, it is the first study to discover and analyze the impact of *Caulobacter* and *Novosphingobium* on the outcomes of CRC. Although studies have reported that microbiome was associated with the prognosis of CRCs, especially Fn, few studies found the direct relationship between other specific bacteria and CRCs. In this study, we used survival data analysis and prognosis-related bacteria to explore the impact of microbiome on patient prognosis by combining it with clinical characteristics and to construct predictive models. Despite the fact that 16sRNA gene sequencing tested CRC tumoral microbiome, more precise sequencing methods, such as metagenomics sequencing and single-cell sequencing, are needed in the future.

This study had several limitations. First, this study is a retrospective study; although there is no difference between the two groups in clinical characteristics, different lifestyles, regions, and dietary habits may lead to microbial changes. Second, the patients came from a single-center study; there were no tumor specimens from other centers that can be used for verification, and as a retrospective study, the conclusions may require further validation from prospective studies. Third, only tumor tissue, not gut microbiota or oral microbiome, was discussed and analyzed. In addition, the impact of fungi or virus was not discussed. We believe that the results presented herein give solid evidence exhibiting the role of the tumoral microbiome in CRC outcomes. In the future, we hope that these data will produce actionable strategies for the implementation of the microbiome to improve CRC outcomes.

In conclusion, this study demonstrates different tumor microbial compositions of CRC patients with different survival times. We also proved that microbiomes may affect the prognosis of tumor patients and can be used as an independent factor to predict the prognosis. Furthermore, the significance of this study is that regulating intra-tumor microbiome during CRC treatment may be important for CRC patients. Measurement of *Caulobacter* and *Novosphingobium* levels may serve as biomarkers for the prognosis of CRC patients, and changing the microbial composition of patients may lead to better prognosis of CRC. Additional studies exploring the relationship between tumoral microbiome, tumoral immune microenvironment, and survival time of patients are needed to further understand the role of the microbiome in CRC prognosis.

## Data availability statement

The original contributions presented in the study are included in the article/**Supplementary Material**. Further inquiries can be directed to the corresponding author.

## Ethics statement

The studies involving human participants were reviewed and approved by Ethics Committee of Tongji Medical College of Huazhong University of Science and Technology (Approval No. 2014-041). The patients/participants provided their written informed consent to participate in this study.

## Author contributions

All authors took part in design of the study protocol, collection, analysis, and interpretation of the data. HL contributed to the conception, designed the study, and critically reviewed the manuscript. BZ made substantial contributions to analysis, interpretation of the data, and had the main responsibility of preparing the manuscript. LS and MC provided the assistance with the sample collection. JZ provided the statistical expertise. DY and TZ critically reviewed the manuscript for important intellectual content. YC provided the helpful discussions and technical support. All authors have reviewed the final version of the manuscript and approve it for publication.
