# Supplementary material for: Caulobacter and Novosphingobium in tumor tissues are associated with colorectal cancer outcomes
Source: Front Oncol. 2023 Jan 27;12:1078296. doi: 10.3389/fonc.2022.1078296 (PMC9911885; doi:10.3389/fonc.2022.1078296)

Supplementary Figure 1. Different clinical characteristics in BL and BS of Novoshpingobium result in distinct overall survival. (A) Overall survival stratified by gut diversity and T stage; (B) Overall survival stratified by gut diversity and N stage; (C) Vessel invasion; (D) Neural invasion. N: Negative; P: positive

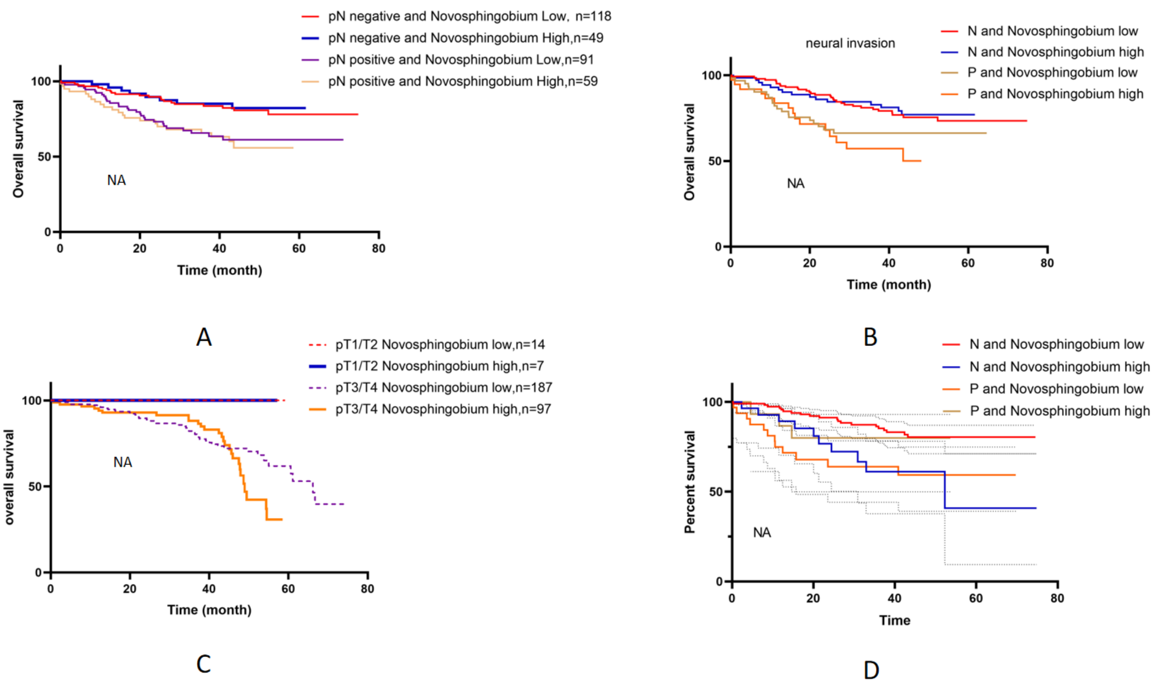

Supplement: Supplementary file 1 [file Image_1.pdf]
